# Supplementary material for: The interactome of intact mitochondria by cross-linking mass spectrometry provides evidence for coexisting respiratory supercomplexes
Source: Mol Cell Proteomics. 2017 Dec 8;17(2):216–32. doi: 10.1074/mcp.RA117.000470 (PMC5795388; doi:10.1074/mcp.RA117.000470)
Supplement: Supplemental Data [file supp_RA117.000470_133922_0_supp_23978_fzffwf.zip › spectra_annotation/mito_spectra_annotation/493-1-3-1-3-1.pdf]

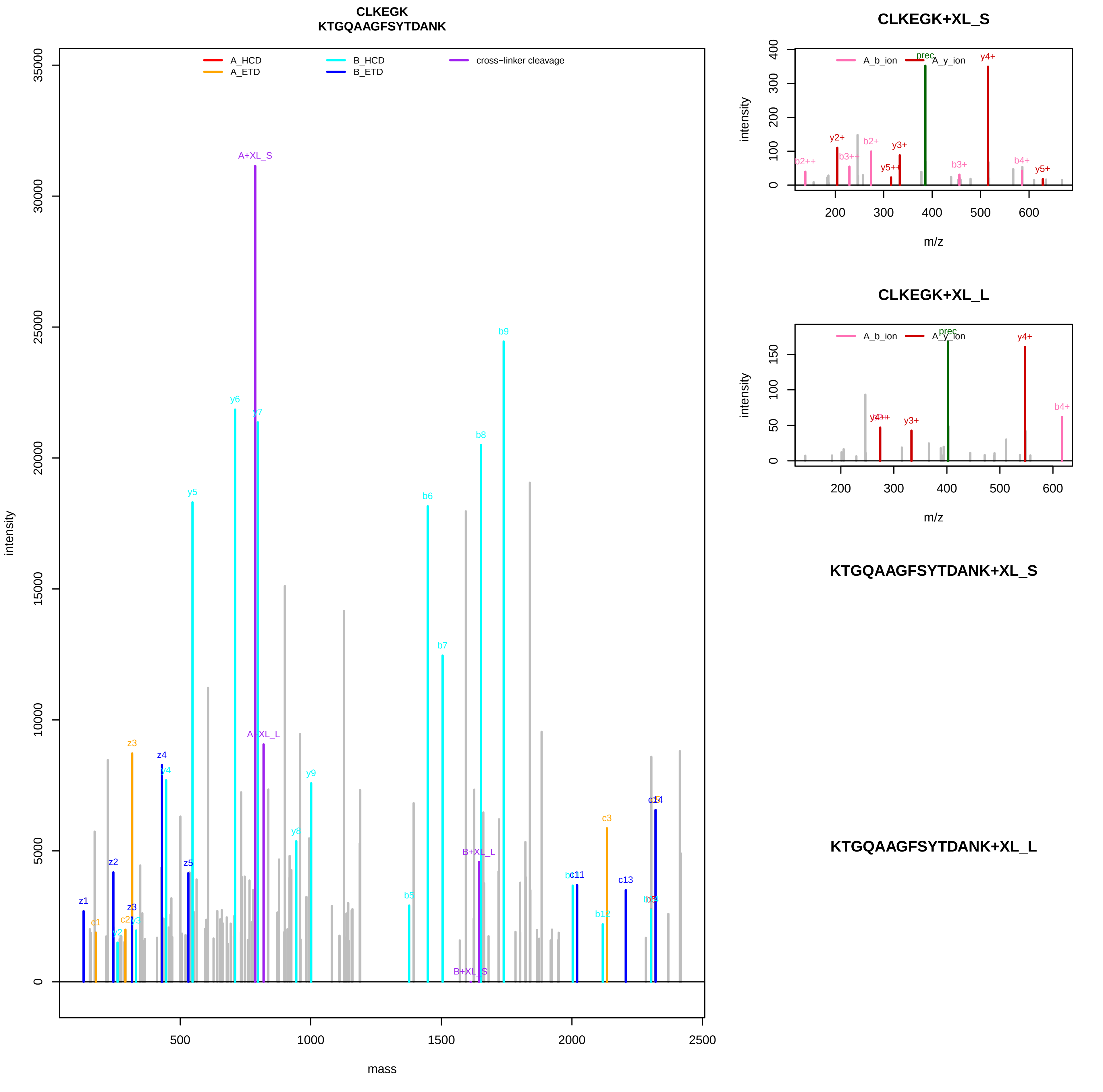
CLKEGK
KTGQAAGFSYTDANK
A_HCD
A_ETD
B_HCD
B_ETD
cross−linker cleavage
A+XL_S
A+XL_L
B+XL_L
B+XL_S
intensity
mass
0
5000
10000
15000
20000
25000
30000
35000
500
1000
1500
2000
2500
z1 c1 y2 c2 z2 z3 y3 z3 z4 y4 z5 y5 y6 y7 y8 y9 b5 b6 b7 b8 b9 c11 b12 c3 c13 c14
CLKEGK+XL_S
A_b_ion
A_y_ion
prec
b2++ y2+ b3++ b2+ y5++ y3+ b3+ y4+ b4+ y5+
intensity
m/z
0
100
200
300
400
200
300
400
500
600
CLKEGK+XL_L
A_b_ion
A_y_ion
prec
y3++ y3+ y4+ b4+
intensity
m/z
0
50
100
150
200
300
400
500
600
KTGQAAGFSYTDANK+XL_S
KTGQAAGFSYTDANK+XL_L
